# Supplementary material for: Frequency Limits of Sequential Readout for Sensing AC Magnetic Fields Using Nitrogen-Vacancy Centers in Diamond
Source: Sensors (Basel). 2023 Aug 31;23(17):7566. doi: 10.3390/s23177566 (PMC10490748; doi:10.3390/s23177566)
Supplement: Supplementary file 1 [file sensors-23-07566-s001.zip › sensors-2536333-supplementary.pdf]

sensors

MDPI

Frequency Limits of Sequential Readout for Sensing AC Magnetic Fields Using Nitrogen-Vacancy Centers in Diamond—Supplementary Material

Santosh Ghimire [1], Seong-Joo Lee [1], Sangwon Oh [1] and Jeong Hyun Shim [1,2,*]

[1] Quantum Magnetic Imaging Team, Korea Research Institute of Standards and Science, Daejeon 34113, Republic of Korea ; santoshgmre@kriss.re.kr (S.G.); sj.lee@kriss.re.kr (S.-j.L.); sangwon.oh@kriss.re.kr (S.O.)

[2] Department of Applied Measurement Science, University of Science and Technology, Daejeon 34113, Republic of Korea

* Correspondence: jhshim@kriss.re.kr

1. Experimental

The optical setup of our system and microwave mixing process in the present experiment are shown in Figure S1. To measure the sensitivity, the diamond is placed between the parabolic concentrator and the microwave loop. The microwave loop, with a diameter 2 mm, is printed on a printed circuit board (PCB). A ring magnet is used to provide the static magnetic field, which is alligned along one of the four axes of NV center. This aligned axis is used to measure the sensitivity. The static magnetic field on the NV center created by the ring magnet is approximately 5.4 mT. The initialization and readout of the NV spin state take place using a 532 nm continuous wave laser. The laser passess through lens L1 to focus on the acousto-optic modulator (AOM). After the AOM, an iris is placed to block the zeroth diffraction order and allow only the first order diffraction beam of the laser to pass through it. Lens L2 is used to demagnify the laser beam. To adjust the polarization plane, a half wave plate (HWP) is used, which hepls to optimize the NV measurement contrast. Lens L3 is used to focus the laser beam on the NV layer. A parabolic concentrator is employed to collect the maximum numbers of photons from NV layer. A long pass filter is placed just above the concentrator, allowing only NV flouriscence to pass through the photodiode. The photodiode detects the NV signal and transmits it to the data acquisition (DAQ) through a transimpedence amplifier (TIA). The TIA is used to amplify the signal and minimize the noise. To apply the oscillating magnetic field, a home-build coil frame is used and placed around the NV sample. The oscillating magnetic field is produced by the function/arbitory waveform generator (SDG 2042X, SIGLENT). A synthesized clock generator (CG635, Standard Research System) is connected to the pulsed blaster, arbitrary waveform generator (AWG), and AC source.

To apply the dynamical decoupling (DD) pulse patterns to the NV ensemble, an arbitory waveform generator (AWG) is used. The DD pulse patterns are saved in the AWG with the help of LabView program. The signals from the RF signal generator (SG384) and AWG are mixed using an RF mixer. The LO, IF, and RF in Figure 1 represents the local oscillator, intermidiate frequency, and radio frequency. After the mixer, a band pass filter (BPF) is employed to block unwanted signals. As the signals are attenuated after mixing, a pre-amplifier (ZRL-3500+, Mini-Circuits) is used to amplify the signal. Two switches are used to create two different paths for the signal; one path allows the contineous wave to pass through, and the other is for pulse generation. After switch 2, the signal passes through a power amplifier and finally reaches the NV sample through the coil antena. The AOM, AWG, AC source, and switches are all connected to the pulsed blaster to generate the desire pulsed patterns.

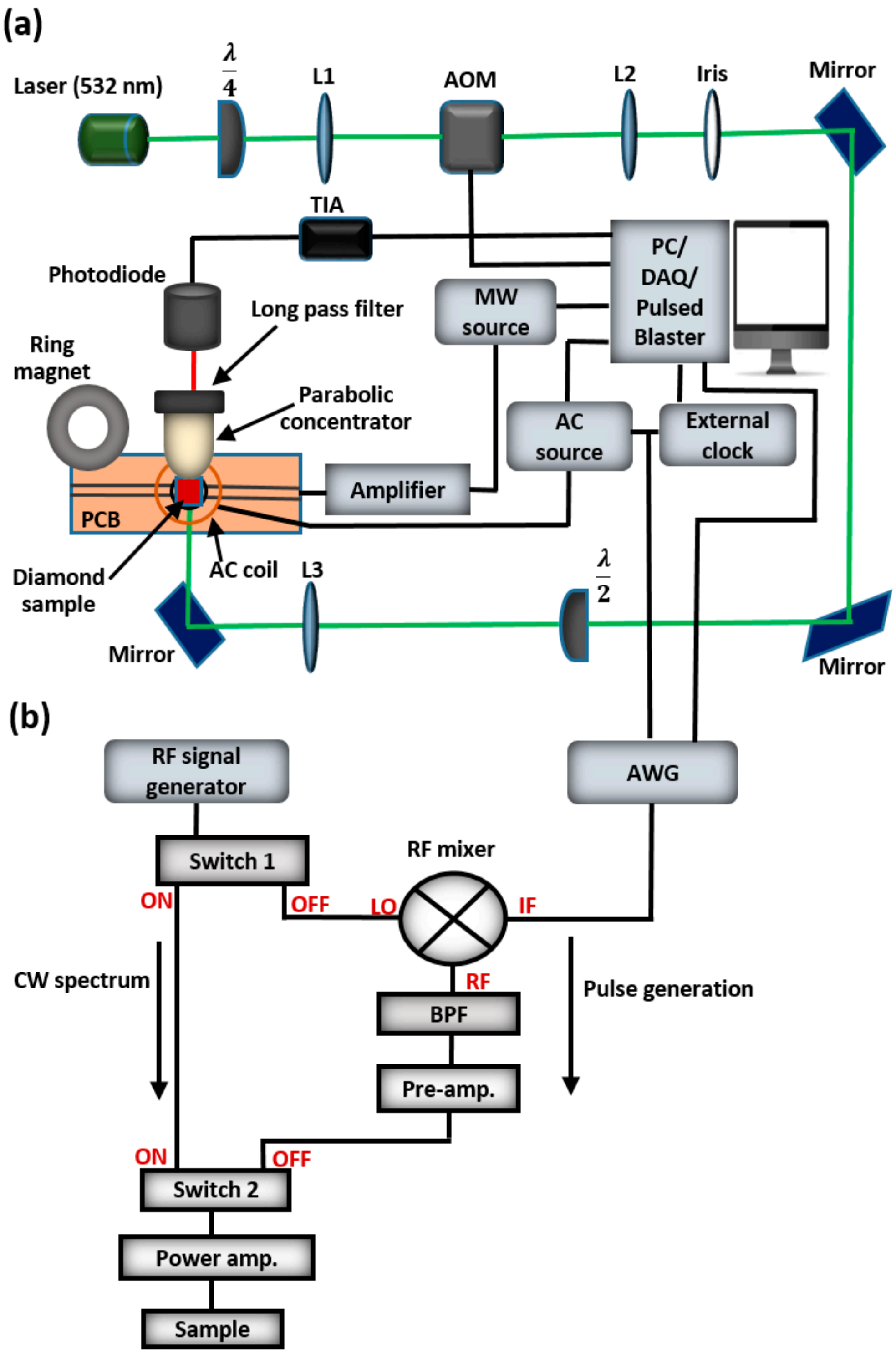

Figure S1. Schematic of (**a**) experimental setup and (**b**) frequency mixing process.

2. Rabi oscillation

We aligned the static magnetic field in one of the four orientations of the NV center and measure the Rabi oscillation to estimate the duration of π pulse, as shown in Figure S2. At the Rabi frequency of 6.3 MHz, the length of π pulse duration is measured to be 80 ns. To perform this measurement, we first initialize the NV spin to zero state using an initial laser pulse. Then, a resonant microwave pulse is applied to drive it to the excited

state, followed by another laser pulse for spin state readout. The duration of the microwave pulse is varied to observe the Rabi oscillation.

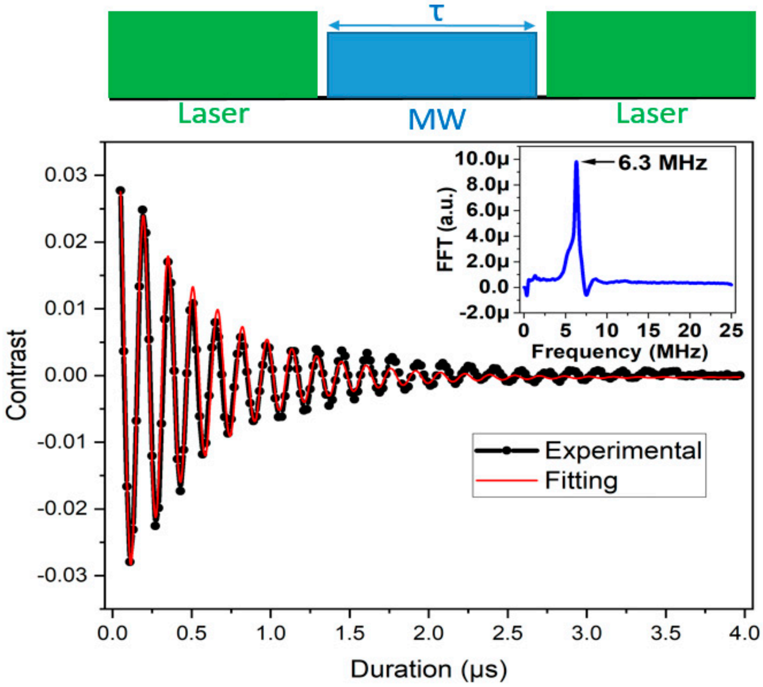

Figure S2. Rabi experiment. The upper part depicts the measurement pulse sequence, and the lower part displays the experimental data with varying microwave (MW) duration upto 4 μs. The red line represents the sine-exponential fitting. The inset shows the FFT fitting data used to estimate the frequency.

3. Tau scan

We applied an external oscillating magnetic field with a frequency of 1 MHz ($f_{ac} = 1/\tau_{ac}$) through the coil antenna. By varying the free precession time between two π pulses (τ) in the XY-4(4) sequence, we detected the applied oscillating magnetic field. When the duration between two π pulses (τ) matches with half of the time period of the external oscillating magnetic field ($\tau_{ac}/2$), a dip appears due to phase accumulation throughout the entire XY-4(4) pulse sequences . In the present experiment, the formation of the dip follows the equation $f_{ac} = (2k + 1)/2\tau(1 + \alpha)$, where $\alpha = \tau_\pi/\tau$ and τ_π is the pulse width of π pulse. In this equation, k = 0 corresponds to the initial time of phase accumulation, and k=1 corresponds to the second phase accumulation time.

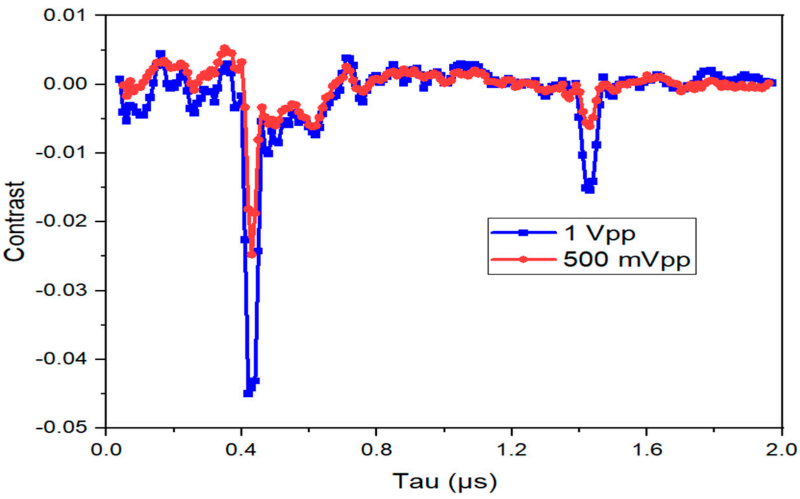

Figure S3. Tau scan. Dips are observed due to the phase accumulation caused by the external oscillating AC signal, as described by the equation $f_{ac} = (2k + 1)/2\tau(1 + \alpha)$.

4. Amplitude of AC magnetic field sweeping

To determine the optimal number of π pulses in the XY-4(N) sequence for measuring AC sensitivity, we sweep the peak-to-peak voltage of the AC magnetic field (V_{pp}) and measure the corresponding contrast change with the V_{pp} as shown in Figure S4. The contrast of NV magnetometry exhibits the periodic oscillations as a function of the V_{pp}. Among the different sequences tested, the curve obtained with the XY-4(4) sequence exhibits a higher slope for a 1 MHz AC source frequency. Therefore, we select the XY-4(4) sequence for sensitivity measurements.

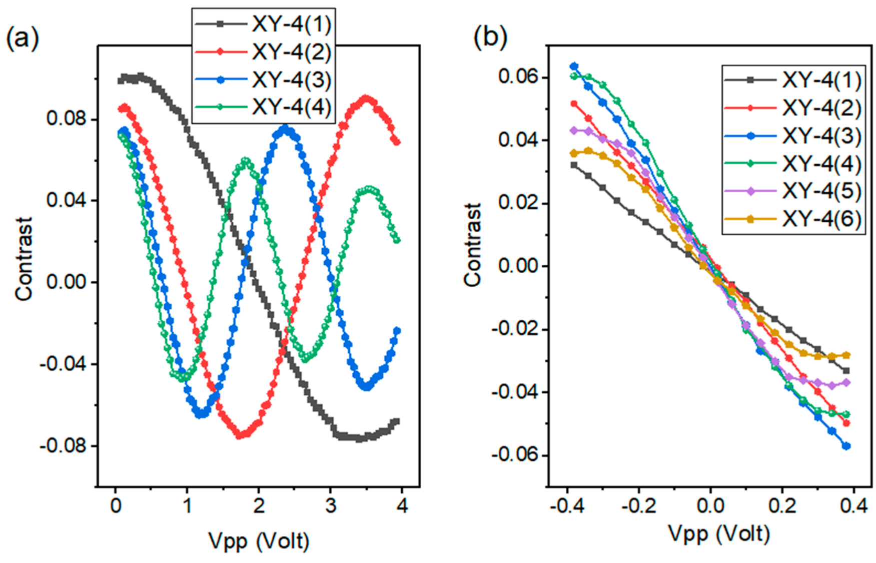

Figure S4. (**a**) The sweeping of the peak-to-peak voltage of AC magnetic field (V_{pp}) to callibrate the sensitivity measurement. (**b**) Zoomed view.

5. T_2 measurement

The tau scan measurement is conducted, and exponential fitting was performed to estimate the decoherence time T_2, as shown in Figure S5. The equation, $y = Ae^{-\left(\frac{N\tau}{T_2}\right)^p} + B$ is used for fitting. This function fits the exponentially decaying envelop and exhibits a characteristic decay time. In the figure, red data points, which seem to be distorted due to external noises, are omitted to perform the exponential fitting. For the XY-4(4) sequence,

the estimated value of T_2 is 16.32 μs. This value is utilized to plot the theoretical curve of sensitivity versus the frequency of the AC source.

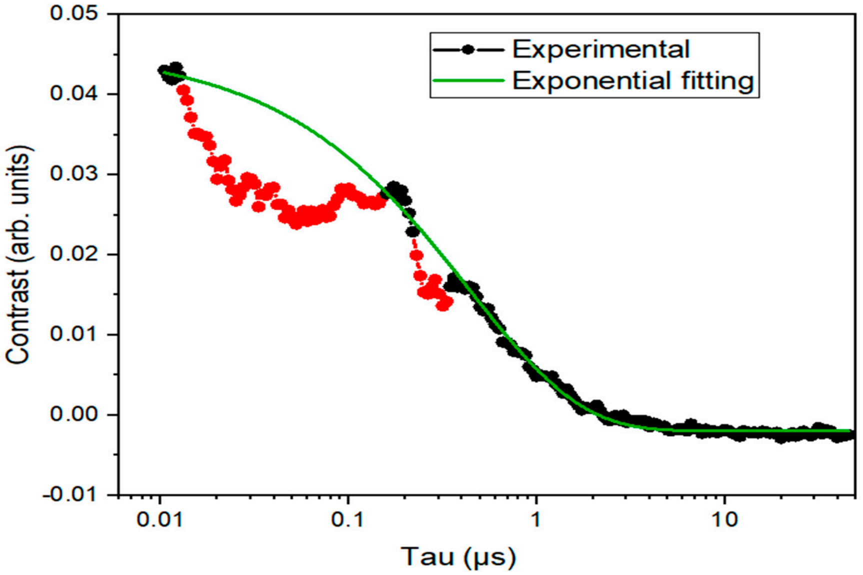

Figure S5. Tau scan to estimate the decoherence time (T_2).

6. AC-field coil current vs frequency

We measured the current flowing through the AC coil by varying the frequency of the AC magnetic field. The AC field was produced using an function generator. The voltage output was kept constant for all frequencies, and the current was measured by a current probe. As the frequency changed from 200 kHz to 5 MHz, the current flow in the coil changed by approximately 11%. For the sensitivity measurement, we calibrated the signal for 1 MHz frequency and included this effect to calculate the sensitivity for other frequencies.

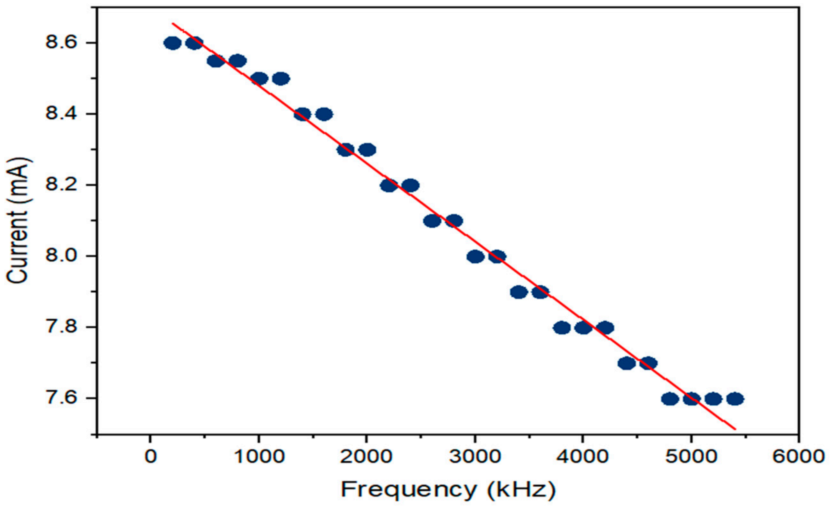

Figure S6. Variation of current as a function of AC source frequency.

7. Derivation of the acquired frequency f_{sens}

Here, we will present the derivation of Equation (3) in the text. The explanation regarding Equation (4) states that when the phase Φ is small, the signal amplitude becomes proportional to Φ. According to Equation (2), $\Phi = \Phi_0 \cos \phi_{ac}$, where ϕ_{ac} is the staring

phase of AC magnetic field. During the SR protocol shown in Figure 2, at the i-th sampling time $t_i = i\,T_{SR}$, the starting phase of the AC field $B_{ac}(t)$ is given as $2\pi f_{ac} \mathrm{i}\delta$. Then, the signal amplitude at t_i, A_i, can be written as

$$A_i = A_o \cos(2\pi f_{ac} \mathrm{i}\delta) = A_o \cos(2\pi f_{sens} \mathrm{i} T_{SR}) \tag{S1}$$

where f_{sens} is the frequency of the signal measured by the SR method. Then, δ can be expressed as

$$\delta = T_{SR} - \bar{n} T_{ac}. \tag{S2}$$

As shown in Figure 2, the integer $\bar{n}$ should satisfy the equation $\bar{n}$ = argmin$_n$($|T_{SR} - nT_{ac}|$). By multiplying $f_{SR} f_{ac}$, $\bar{n}$ can be obtained from the frequencies f_{SR} and f_{ac} as $\bar{n}$ = argmin$_n$($|f_{ac} - nf_{SR}|$). From Equations (S1) and (S2), $f_{ac}(T_{SR} - \bar{n}T_{ac}) = f_{sens} T_{SR}$. Then, we can obtain Equation (4) as below

$$f_{sens} = f_{ac}\left(1 - \bar{n}\frac{T_{ac}}{T_{SR}}\right) = f_{ac} - \bar{n} f_{SR}. \tag{S3}$$
